# Special Issue: Selected Papers from Experimental Stress Analysis 2020

**DOI:** 10.3390/ma14051136

**Published:** 2021-02-28

**Authors:** Lenka Kunčická, Radim Halama, Martin Fusek

**Affiliations:** 1 Institute of Physics of Materials, Czech Academy of Sciences, 61662 Brno, Czech Republic; kuncicka@ipm.cz; 2 Faculty of Mechanical Engineering, VŠB-Technical University of Ostrava, 70800 Ostrava, Czech Republic; martin.fusek@vsb.cz

The contemporary way of living brings about increasing demands on materials used in our everyday lives. Challenging requirements are put on the performance of modern metallic, as well as non-metallic, materials throughout commerce and industry. These requirements are not only from the viewpoint of their lifetime and durability, but also in the context of their user friendliness and safety. A great example of the importance of human safety in everyday life is presented by the published paper studying the effects of a collision involving a pedestrian with a tramway vehicle [1].

A great method that is of help during the research and development of innovative materials and components is the finite element method (FEM). Based on numerical computations performed with reliable models of real assemblies with optimized boundary conditions [2], FEM is not only useful for the thorough testing of high-level properties of durable metallic materials and components [3,4], but is also helpful in numerous other applications. Such applications include the abovementioned investigation of the impacts of a pedestrian/tram collision [1], simulations of the performance of bio-applicable components and materials [5,6,7], and in solving challenging industrial tasks [8,9].

However, the greatest impact on the successful and satisfactory performance of innovative components has been on the type of the material used, and the applied production and processing technologies. In addition to conventional alloys [10] and commercially pure elements [11], modern materials also include light and durable metallic composites [12], carbon tube reinforced polymers (CTRP) [13], and thermoplastic composites [14]. Additive manufacturing (or 3D printing) of powders, including methods such as selective laser melting (SLM) [3], has recently gained a great deal of attention. However, conventional production technologies, such as casting and welding [10,15], are still popular, especially for specific applications (large components or semi-products). Nevertheless, conventional (rolling, forging, etc. [16]), as well as unconventional (severe plastic deformation (SPD) methods, rotary swaging, etc. [17,18]) processing methods are applicable for both types of semi-products, i.e., conventionally produced and 3D printed. Optimized selection of the methods and conditions of processing can greatly enhance the performance and properties of virtually any semi-product.

Last but not least, research of innovative materials and components goes hand in hand with the development of modern testing methods, advantageously non-destructive ones, some of which are also of interest to the presented Special Issue. Popular methods are those based on optical systems, such as the PhotoStress and digital image correlation (DIC) methods [19], or the acoustic emission method based on transferring acoustic signals through the tested material [20]. Neutron diffraction is also among the top-level non-destructive testing methods [21,22].
